# Cytotoxicity, Cell Cycle Arrest, and Apoptosis Induction Activity of Ethyl-p-methoxycinnamate in Cholangiocarcinoma Cell

**DOI:** 10.31557/APJCP.2020.21.4.927

**Published:** 2020-04

**Authors:** Phunuch Muhamad, Luxsana Panrit, Wanna Chaijaroenkul, Kesara Na-Bangchang

**Affiliations:** 1 *Drug Discovery and Development Center, Office of Advanced Science and Technology, *; 2 *Center of Excellence in Pharmacology of Malaria and Cholangiocarcinoma, *; 3 *Graduate Program in Bioclinical Sciences, Chulabhorn International College of Medicine, Thammasat University, Pathumthani, Thailand. *; 4 **gchang** ^**1,2,3**^ *

**Keywords:** Apoptosis, cell cycles arrest, chloangiocarcinoma, ethyl-p-methoxycinnamate (EPMC), p-glycoprotein

## Abstract

**Objective:**

To investigate cytotoxic activity of ethyl-p-methoxycinnamate (EPMC) including its effect on p-glycoprotein (multidrug resistance-1: *mdr-1* gene) in human cholangiocarcinoma cell.

**Methods:**

Cytotoxic activity of EPMC against human cholangiocarcinoma (CL-6), fibroblast (OUMS-36T-1F), and colon cancer (Caco-2) cell lines were assessed using MTT assay. Selectivity index (SI) was determined as the ratio of IC_50_ (concentration that inhibits cell growth by 50%) of EPMC in OUMS-36T-1F and that in CL-6 cell. Cell cycle arrest and apoptosis in CL-6 cells were investigated by flow cytometry and fluorescent microscopy. Effect of EPMC on *mdr-1* gene expression in CL-6 and Caco-2 was determined by real-time PCR.

**Results:**

The median (95% CI) IC_50 _values of EPMC in CL-6, OUMS-36T-1F, and Caco-2 were 245.5 (243.1-266.7), 899.60 (855.8-966.3) and 347.0 (340.3-356.9) µg/ml, respectively. The SI value of the compound for the CL-6 cell was 3.70. EPMC at IC_50_ inhibited CL-6 cell division and induced apoptosis compared to untreated control. EPMC exposure did not induce *mdr-1* gene expression in both CL-6 and Caco-2 cells.

**Conclusion:**

The results suggest the potential role of EPMC in cholangiocarcinoma with a low possibility of drug resistance induction.

## Introduction

Cholangiocarcinoma is the bile duct malignancy, which the highest incidence has been reported in the population living in the North-Eastern region of Thailand (Kirstein and Vogel, 2016, Xia et al., 2015). The major risk factors are ingestion of *Opisthorchis viverrini* metacercaria contaminated raw cyprinoid fish, family history of cancer, and liquor consumption (Hughes et al., 2017; Kamsa-ard et al., 2018). Most cholangiocarcinoma cases are diagnosed when the disease progresses to late stage and must receive treatment by chemotherapeutic drugs. The 5-Fluorouracil (5-FU) is the first-line drug for cholangiocarcinoma but drug resistance results in reduced treatment efficacy (Konstantinidis et al., 2016). 

*Kaempferia galanga* Linn. (known as Proh-hom in Thai) exhibits several potential medicinal properties such as analgesic (Sulaiman et al., 2008), anti-inflammatory (Sulaiman, et al., 2008), carminative (Preetha et al., 2016) and sedative activities (Huang et al., 2008). Consumption of this plant promotes heat production that enhances recovery of sickness (Nishidono et al., 2017). Ethyl-p-methoxycinnamate (EPMC), the major active compound isolated from *K. galanga* L., exhibits nematicidal (Jae-Kook et al., 2011), antibacterial (Lakshmanan et al., 2011), anti-inflammatory (Umar et al., 2014), and antineoplastic activities (Umar et al., 2018) including inhibition of hepatocellular carcinoma (Liu et al., 2010) and blood vessel formation (He et al., 2012). Based on the information, EPMC might also have anticancer activity in cholangiocarcinoma. Apoptosis and cell cycle arrest activities are major parameters to evaluate anticancer properties of potential candidate compounds (Czarnomysy et al., 2018; Huang et al., 2018). However, potential drug resistance induction by these compounds should be an issue of concern in the development of novel anticancer agents (Huang et al., 2018). Chemoresistance often caused by overexpression of *mdr-1* gene that encodes p-glycoprotein or multidrug resistance protein 1 (MDR1) (Hodges et al., 2011). The protein has a role as drug transporter which can pump out several substrate anticancer drugs such as 5-ﬂuorouracil (5-FU), cisplatin, doxorubicin, methotrexate, tamoxifen, vinblastine, and vincristine and be inducible by several compounds (Chen et al., 2016; Hu et al., 2016). *Mdr-1* gene overexpression found in many tumor cells including leukaemia (Yague et al., 2003), colon cancer (Hu, et al., 2016), breast cancer (David et al., 2004), hepatocellular carcinoma (Grude et al., 2002) and cholangiocarcinoma (Marin et al., 2018).

Treatment failure in cholangiocarcinoma patients promotes the requirement of novel anticancer agents to replace the commonly used anticancer drugs. Therefore, the study aimed to investigate cytotoxic activity of EPMC in in various human cell lines and its effect on drug resistance.

## Materials and Methods

*Cell line and culture*

The cell lines used in this study were human cholangiocarcinoma, CL-6 (kindly provided by Associate Professor Dr. Adisak Wongkajornsilp, Faculty of Medicine, Siriraj Hospital, Mahidol University, Thailand), human fibroblast (OUMS-36T-1F: Japanese Collection of Research Bioresources (JCRB) cell bank, Japan), and human colon cancer (Caco-2: American Type Culture Collection (ATCC), USA). All were maintained in culture according to the previously described procedures (Chaijaroenkul et al., 2011; Kotawong et al., 2018; Sumsakul and Na-Bangchang, 2015). The CL-6 and OUMS-36T-1F cell lines were cultured in RPMI 1640 (Gibco, NY, USA) and Dulbecco’s Modified Eagle Medium (DMEM; Gibco, NY, USA), respectively, with supplement of 10% fetal bovine serum (FBS: Gibco, NY, USA) and 100 U/ml antibiotic-antimycotic (Gibco, NY, USA). The Caco-2 cell line was cultured in Minimum Essential Medium (MEM: Gibco, NY, USA) containing 1 mM sodium pyruvate (Gibco, NY, USA), 20% FBS (Gibco, NY, USA), and 100 U/ml antibiotic-antimycotic (Gibco, NY, USA). All cell lines were grown under 37 ^o^C and 5% CO_2_ atmosphere until reaching 80% confluency and were trypsinized with 0.25% trypsin-EDTA (Gibco, NY, USA). Cell viability was examined under the light microscope after staining with 0.4% trypan blue (Gibco, NY, USA). 

*Cytotoxicity assay*

Cytotoxic activity of ethyl-p-methoxycinnamate (EPMC: TCI, Oxford, UK) and 5-fluorouracil (5-FU: FUJIFILM Wako Pure Chemical Corp., Osaka, Japan) against all cell lines was determined according to the previously described method (Chaijaroenkul et al., 2011). The CL-6 (1.0×10^4^ cells/well), OUMS-36T-1F (1.0×10^4 ^cells/well) and Caco-2 cells (1.5×10^4^ cells/well) cells were seeded onto a 96-well plate and incubated overnight at 37 ^o^C in 5% CO_2_ atmosphere. The cells were exposed to EPMC for 48 hours, and cell viability was examined using MTT assay (3, [4, 5-dimethylthiazol-2-yl]-2, 5-diphenyltetrazolium bromide, Molecular Probes, OR, USA). DMSO (100 µl, VWR, PA, USA) was added in each well to dissolve the formazan crystal from the reaction, and color intensity was measured by Varioskan Flash microplate reader (Thermo, MA, USA) at the wavelength of 540 nm. Percentage of cell viability was calculated as follow: 

Cell viability (%) = (OD of treated cells/ OD of control cells) × 100

Analysis of concentration-response curve of EPMC was performed using CalcuSyn™ v2.11 software (Biosoft, Cambridge, UK) to obtain IC_25_, IC_50_ and IC_90 _(concentration that inhibits cell growth by 25%, 50%, and 90%, respectively). Selectivity index (SI) was calculated as the ratio of the IC_25_, IC_50_, and IC_90_ of EPMC against normal cell (OUMS-36T-1F) and cholangiocarcinoma cell (CL-6).

*Wound healing assa*y

Wound healing assay was performed according to the method of Pinto and Ramenzoni with modification (Pinto BI et al., 2018, Ramenzoni et al., 2017). The CL-6 cells (1.0×10^4^ cell/well) were seeded onto a 96-well plate and incubated overnight at 37 ^o^C under 5% CO_2_ atmosphere. The cell monolayers were scratched using a 10 µl pipette tip, and cell debris was removed by rinsing with 1xPBS. The cells were exposed to EPMC at IC_25_ and IC_50_ concentrations for 24 and 48 hours; control cells were exposed to complete RPMI 1640 medium. The images of the scratched areas were examined under the inverted microscope (40x magnification) before EPMC exposure, and at 24 and 48 hours after exposure. 

*Cell cycle arrest investigation*

The CL-6 cell line was grown overnight in a 6-well plate (5×10^5^ cells/well) before exposing to EPMC at the IC_25_ and IC_50_ concentrations for 12, 24, and 48 hours. Cells were harvested by trypsinization, and DNA content was stained by fluorescence dye according to the manufacturer’s protocol (BD Cycletest^TM^ Plus DNA Reagent Kit, BD Biosciences, CA, USA). Briefly, cell pellets were collected through centrifugation at 250 *x *g for 5 minutes and incubated with the provided reagent before staining with propidium iodide (PI). Cell number and copy number of DNA were determined using FACTverse flow cytometer (BD Biosciences, CA, USA) at 278 energy voltage. The experiment was performed in triplicate, and cell development to G0/G1, S and G2/M phases in tested samples were compared to control.

*Apoptosis investigation*

The CL-6 cells (5×10^5^ cells) were grown overnight in a 25 cm^2^ filter flask. Cells were harvested by trypsinization after exposure to EPMC at the IC_25_ and IC_50 _concentrations for 24 and 48 hours. The apoptotic protein marker phosphatidylserine and intracellular DNA content were stained by fluorescence dye according to the manufacturer’s protocol (BD Pharmingen^TM^ FITC Annexin V Apoptosis Detection Kit, BD Biosciences, CA, USA). The numbers of both living and apoptotic (early and late) cells were determined by FACTverse flow cytometer (BD Biosciences, CA, USA) at 281 and 278 energy voltages for FITC and PI, respectively. The experiment was performed in triplicate. 

The apoptotic effect of EPMC on CL-6 cell line was also investigated based on caspase 3/7 expression (Panrit et al., 2018). The cells (3.5×10^4^ cells/well) were seeded in Corning™ Falcon™ Chambered Cell Culture Slides (Corning, NY, USA) and incubated overnight at 37^o^C under 5% CO_2_ atmosphere before exposing to EPMC at IC_25_ and IC_50_ concentrations for 24 and 48 hours. The apoptotic proteins caspase 3/7 were stained with fluorescence CellEvent™ Caspase-3/7 Green detection assay (Thermo Fisher Scientific, NY, USA) and cells with caspase-3/7 expression were examined under ZOE fluorescent microscope (Bio-rad, CA, USA) using 40x objective lens. The green stained apoptotic cells were observed in 10 fields and the ratio of cell apoptosis was estimated as follow:

Caspase 3/7 expression (%) = (Mean fluorescence-stained cells/Mean of total cell number) × 100

*Mdr-1 gene expression*

The CL-6 cells (5.0 × 10^5^ cells) were grown in a 6-well plate for 24 hours before exposing to EPMC (0.08, 0.8, and 8 µg/ml). The Caco-2 cells were seeded onto a 6-well plate at the same number for 48 hours before exposing to EPMC (0.2, 2, and 20 µg/ml). Following 24 and 48 hours incubation, RNA contents of both cells were collected using Trizol^TM^ reagent (Invitrogen, NY, USA). The RNA was then converted to cDNA according to the manufacturer’s protocol (SuperScript™ III Reverse Transcriptase kit, Invitrogen, NY, USA). Gene expression was determined by SYBR green real-time PCR using MDR1-F: GTCTTTGGTGCCATGGCCGT, MDR1-R: ATGTCCGGTCGGGTGGGATA for *mdr-1* gene (target gene) and GAPDH-F: CAACAGCCTCAAGATCATC AGC, GAPDH-R: TTCTAGACGGCAGGTCAGGTC for gapdh gene (reference gene or housekeeping gene). The cDNA template was amplified according to the following conditions: pre-denaturation at 95 ^o^C for 5 min, denaturation at 95 ^o^C for 15 sec, and annealing at 60 ^o^C for 1 min with fluorescence intensity detection. The melting curve was plotted every 0.5^o^C until reaching 95 ^o^C (Chaijaroenkul et al., 2011). *Mdr-1* gene expression level was calculated relative to gapdh gene expression and normalized with H0 sample according to delta-delta C_t_ method (Sumsakul and Na-Bangchang, 2015) as follows: 

C_t_ (tested) = [C_t_ (*mdr-1*, tested) - C_t_ (*gapdh*, tested)]

C_t_ (control) = [C_t_ (*mdr-1*, control) - C_t_ (*gapdh*, control)]

C_t_ = C_t_ (tested or control) - C_t_ (H0)

Relative expression = 2^-^^δδ^^Ct^

*Data analysis*

Each experiment was performed in triplicate. Cytotoxic activity of EPMC against CL-6, OUMS-36T-1F and Caco-2 cells was expressed as median (95% CI) values of IC_25_, IC_50_, and IC_90_. Quantitative data are presented as median (95% CI) values. Difference between the two independent quantitative groups was determined using Mann Whitney U test at a statistical significance level of α = 0.05.

**Figure 1:**
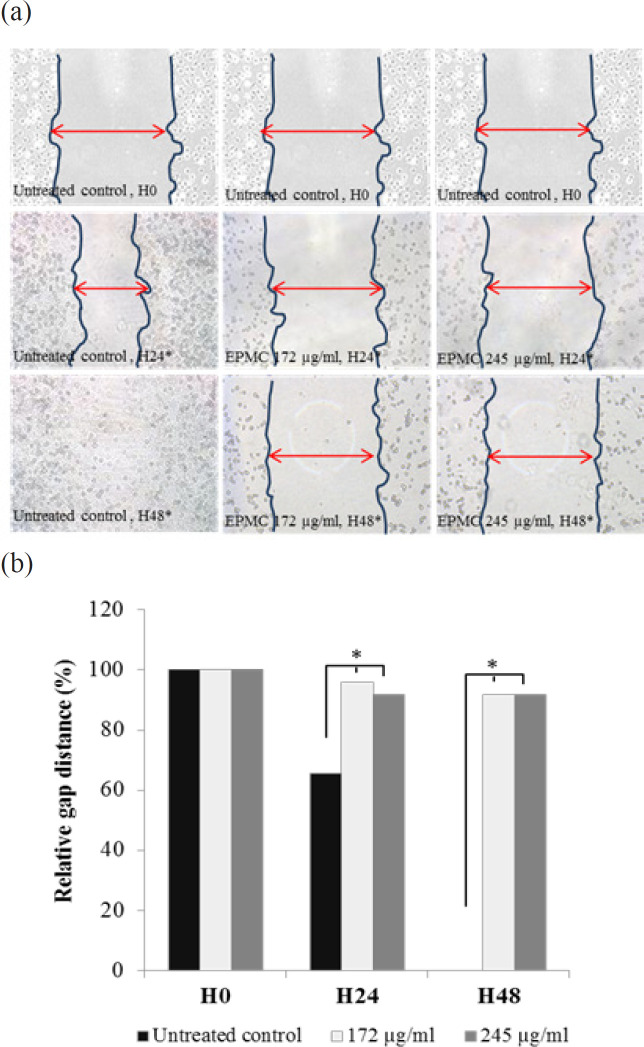
Cell Migration Activity Determined by Wound Healing Assay in CL-6 Cell Following Exposure to EPMC at IC_25_ (172 µg/ml) and IC_50_ (245 µg/ml) Concentrations for 24 and 48 Hours. (a) Inhibition effect presented as cell gaps distance and (b) relative gap distance. (*) showed statistically significant wound healing inhibition compared to untreated control. EPMC, ethyl p-methoxycinnamate; H0, 0 hours; H24, 24 hours; H48, 48 hours

**Table 1:** Inhibitory Concentrations (ICs) of EPMC and 5-FU Against CL-6, OUMS-36T-1F, and Caco-2 Cell Lines Data are Presented as Median (95% CI) Values

Cell	Compound	Inhibitory concentration, µg/ml		
		IC_25_	IC_50_	IC_90_
CL-6	EPMC	172.3	245.5	498.3
		(120.6-188.2)	(243.1-266.7)	(498.9-535.5)
	5-FU	9.76	120.6	1489.7
		(8.95-9.84)	(117.9-128.0)	(1411.4-1830.3)
OUMS-36T-1F	EPMC	702.1	899.6	1556.5
		(634.5-750.4)	(855.8-966.3)	(1476.8-1602.2)
Caco-2	EPMC	226.2	347.0	816.5
		(221.1-226.9)	(340.3-356.9)	(805.7-883.2)
Selectivity Index (SI)				
OUMS-36T-1F/CL-6	EPMC	4.1	3.7	3.1

EPMC, ethyl p-methoxycinnamate; 5-FU, 5- Fluorouracil; IC, Inhibitory concentrations

**Figure 2:**
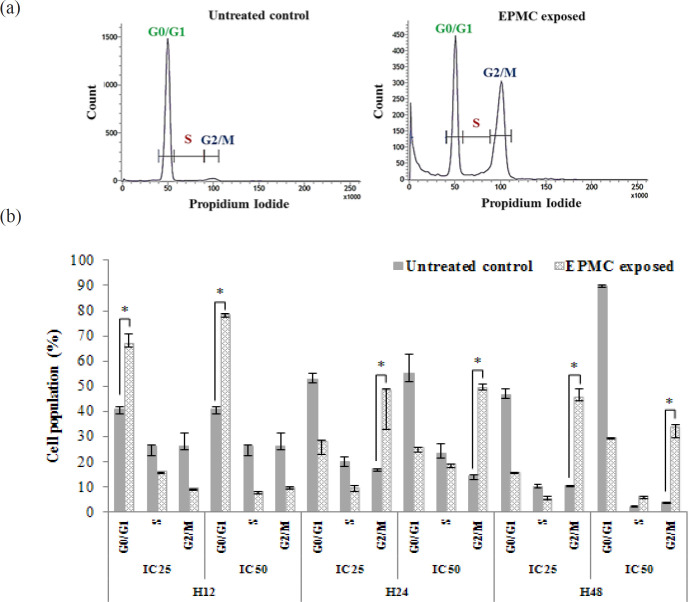
Cell Cycle Analysis of CL-6 Cell Following Exposure to EPMC at IC_25 _(172 µg/ml) and IC_50_ (245 µg/ml) Concentrations for 12, 24, and 48 Hours. (a) Cell cycle histogram of CL-6 cell untreated control and (b) EPMC-exposed (at IC_50_) for 48 hours and cell cycle distribution of CL-6 cell population following exposure to EPMC at IC_25_ and IC_50_ for 12, 24 and 48 hours. Data are presented as median (95% CI) and (*) showed a significant increase of cell population after EPMC exposure compared to untreated control. EPMC, ethyl p-methoxycinnamate; H12, 12 hours; H24, 24 hours; H48, 48 hours

**Figure 3:**
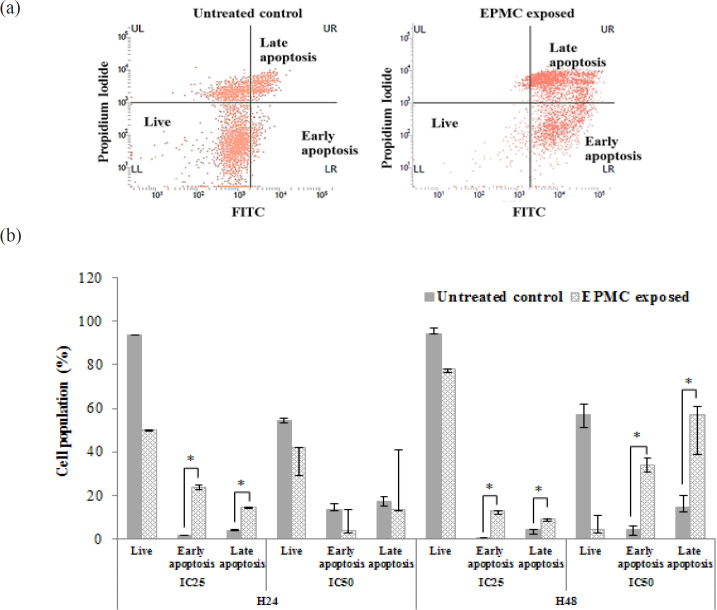
Apoptosis Investigation of CL-6 Cell Following Exposure to EPMC at IC_25_ (172 µg/ml) and IC_50_ (245 µg/ml) Concentrations for 24 and 48 Hours. (a) Scatter plots of apoptosis in the untreated control and EPMC-exposed (at IC_50_) CL-6 cell for 48 hours and (b) apoptosis degree of CL-6 cell population following EPMC exposure at IC_25_ and IC_50_ for 24 and 48 hours. Data are presented as median (95% CI) and (*) showed a significant increase in cell population after EPMC exposure compared to untreated control. EPMC, ethyl p-methoxycinnamate; H24, 24 hours; H48, 48 hours

**Figure 4:**
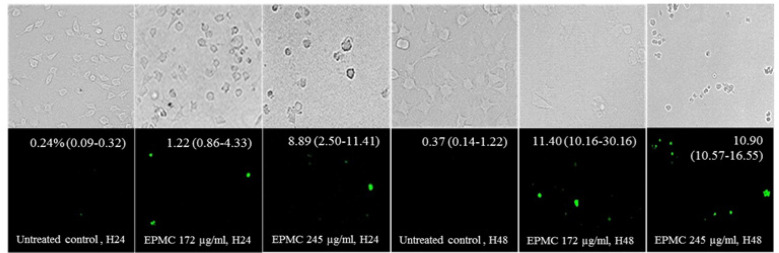
Apoptosis Activity Through Induction of Caspase 3/7 Expression in CL-6 cells Following Exposure to EPMC at IC_25_ (172 µg/ml) and IC_50_ (245 µg/ml) Concentrations for 24 and 48 Hours. The upper row represents cell morphology changing under bright field observation, and lower row represents the green dot of caspase 3/7 expression cells at the relevant position by using the fluorescence microscope. Percentages of caspase 3/7 are presented as median (95% CI). EPMC, ethyl p-methoxycinnamate

**Figure 5:**
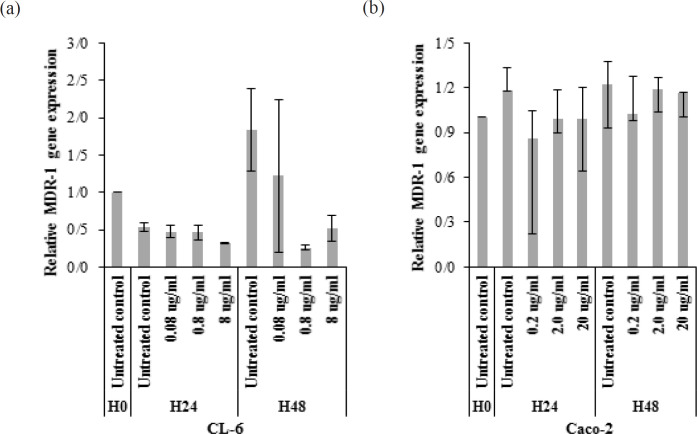
Effect of EPMC on mdr-1 Gene Expression in (a) CL-6 and (b) Caco-2 Cells Following Exposure to the Compound for 24 and 48 Hours. Data are presented as median (95% CI). EPMC, ethyl p-methoxycinnamate; H24, 24 hours; H48, 48 hours

## Results

*Cytotoxic activity of EPMC against CL-6, OUMS-36T-1F, and Caco-2 cells*

Growth inhibitory effects of EPMC and/or 5-FU on CL-6, OUMS-36T-1F, and Caco-2 cell lines expressed as IC_25_, IC_50_, and IC_90_ are summarized in Table 1. The potency of activity of EPMC varied with concentrations. The potency of activity based on IC_50_ was about 50% of 5-FU. The SI value of EPMC at the three concentrations ranged from 3.1 to 4.1. The cytotoxic activity of EPMC was also investigated in the Caco-2 cell line to identify optimal concentration to be used in the *mdr-1* gene induction experiment. The concentration that produced 100% cell viability was used as the maximum concentration for investigation of *mdr-1* gene expression in CL-6 and Caco-2 cell lines. 

*Effect of EPMC on cell migration*

Wound healing assay was performed to determine the effect of EPMC on CL-6 cell migration. CL-6 cell migration was inhibited after exposure to EPMC at both IC_25_ (172 µg/ml) and IC_50_ (245 µg/ml) concentrations for 24 and 48 hours (Figure 1). 

*Effect of EPMC on cell cycle arrest*

The effect of EPMC on CL-6 cell cycle arrest (G0/G1, S, and G2/M) is shown in Figure 2. Following 12 hours of EPMC exposure, CL-6 cell division was obstructed at the G0/G1 phase. Percentage of cell population at this phase was significantly higher than control cells after exposing to EPMC at both IC_25_ and IC_50_ concentrations compared with control [median (95% CI): 66.49 (65.77-70.68)% vs. 40.71 (39.24-41.69)% (p=0.05), and 77.79 (77.55-78.92)% vs. 40.71 (39.24-41.69)% (p=0.05), respectively. 

EPMC exposure for 24 and 48 hours significantly inhibited CL-6 cell division at G2/M phase. At 24 hours, percentage of cell population was significantly higher after exposing to EPMC at both IC_25_ and IC_50_ concentrations compared with control [median (95% CI): 47.92 (32.68-48.78)% vs. 17.03 (16.39-17.27)% (p=0.05), and 48.86 (48.59-50.93)% vs. 14.52 (12.73-14.92)% (p=0.05). At 48 hours, percentage of cell population was significantly higher after exposing to EPMC at both IC_25_ and IC_50_ concentrations [median (95% CI): 45.08 (44.44-48.73)% vs. 10.45 (10.16-10.46)% (p=0.05), and 33.35 (29.47-34.80) vs. 3.91 (3.25-4.08)% (p=0.05).

*Effect of EPMC on apoptosis induction*

Investigation of cell apoptosis using flow cytometer can classify cell population as living, early apoptotic, and late apoptotic cells by FITC-Annexin V and propidium iodide (PI) staining (Figure 3a). EPMC exposure for 48 hours at IC_25_ and IC_50_ concentrations significantly induced both early and late apoptosis. Percentage of early cell apoptosis was significantly higher after exposing to EPMC at both concentrations compared with control [median (95% CI): 12.82 (11.37-12.86)% vs. 0.58 (0.47-0.88)% and 33.83 (30.78-37.04)% vs. 4.54 (1.99-5.80)%] (p=0.05). In addition, percentage of late cell apoptosis was also significantly higher after exposing to EPMC at both concentrations compared with control [median (95% CI): 8.98 (8.25-9.31)% vs. 4.49 (2.23-4.66)% and 56.83 (38.97-60.86% vs. 14.45 (12.31-20.08)% (p=0.05). At the end of 24 hours exposure, however, significant induction of both early and late stages cell apoptosis was found only at IC_25_ for [median (95% CI): 23.67 (22.50-24.84)% vs. 1.85 (1.76-1.94)% and 14.39 (13.97-14.80)% vs. 4.12 (4.07-4.17)% (p=0.05). 

Apoptotic effect of EPMC in CL-6 cells was also confirmed with caspase 3/7 expression. EPMC exposure at IC_25_ and IC_50_ concentrations for 24 and 48 hours induced apoptosis in a time- and concentration-dependent manners. Percentages of apoptotic cell [median (95% CI)] after EPMC exposure at the IC_25_ and IC_50_ concentrations and control cells for 24 hours were 1.22 (0.86-4.33)%, 8.89 (2.50-11.41)% and 0.24 (0.09-0.32)%, respectively. Cell apoptosis population was significantly increased after 48 hours of treatment [11.40 (10.16-30.16)% and 10.90 (10.57-16.55)%, respectively, compared with 0.37 (0.14-1.22)% in control cells] (Figure 4). 

*Effect of EPMC on mdr-1 gene expression*

The *mdr-1* gene was not significantly upregulated in CL-6 and Caco-2 cells following exposure to EPMC (0.08, 0.8, and 8 µg/ml and 0.2, 2, and 20 µg/ml, respectively) for 24 and 48 hours, compared to control sample (p>0.05) (Figure 5). Decreasing of *mdr-1* gene expression was observed in all compound exposed CL-6 cells and conferred to* mdr-1* expression inhibition activity of EPMC.

## Discussion

The potential anticancer activity of *K. galanga* L. crude extract has previously been reported in the CL-6 cell (Amuamuta et al., 2017; Mahavorasirikul et al., 2010). However, the study about the anticancer activity of the isolated compound, EPMC, in cholangiocarcinoma cell still limited. Investigation of EPMC as a promising anticancer in CL-6 cell line revealed its biological activities i.e.; cytotoxicity, wound healing inhibition, apoptosis induction, cell cycle arrest without *mdr-1* gene expression induction. 

The potency of activity of EPMC in CL-6 cell varied with concentrations as its IC_25_, IC_50_, and IC_90_ was 172.3, 245.5, and 498.3 µg/ml, respectively (Table 1). The weak cytotoxic activity of EPMC against CL-6 cells (about 50% of 5-FU) is explained by the ester functional group containing chemical structure which results in weak interaction with other cellular components (Sakagami et al., 2017; Soult, 2018). The selectivity to CL-6 cell was 3.7 times compared to a normal cell, OUMS-36T-1F (Table 1). It is noted for the variable activity, and selectivity of EPMC reported in this study compared with the previous study against the same cell lines (Amuamuta, et al., 2017). In the previous study, the IC_50_ (median [95% CI]) and selectivity index of EPMC were shown to be 49.19 (48.16-52.29) μg/ml and 2.09, respectively. The potency of activity of this active compound was about 1.3 times of the crude ethanolic extract (median IC_50_ 64.2 µg/ml) (Amuamuta, et al., 2017). The anticancer activity of EPMC could be improved when combined with other compounds as the components of *K. galanga *L. such as kaempferol or kaempferide. This remains to be confirmed.

EPMC at IC_25_ and IC_50_ was found to inhibit wound healing in CL-6 cell line after 24 and 48 hours of exposure. This suggests the potential role of the compound to interrupt cell migration and metastasis. Results of the previous toxicity study showed that *K. galangal *L. extract exhibited promising anti-cholangiocarcinoma activity in CL6-xenografted nude mice as determined by significant inhibitory activity on tumor growth and lung metastasis, as well as prolongation of survival time (Amuamuta, et al., 2017).

Several drugs or compounds produce cytotoxic effect through the initiation of cell death mechanisms such as loss of function of essential organelles, apoptosis activation, and cell cycle arrest (Iorga et al., 2017; Sharifi-Rad et al., 2017). Results of the current study suggest that induction of cell cycle arrest and apoptosis are essential anti-cholangiocarcinoma action of EPMC. To determine action in cell cycle arrest, the phases of cell growth in normal condition has been compared with the EPMC exposed sample. CL-6 cell required 24 hours to complete cell division cycle and remained at G0/G1 phase as well as 1 copy or 2n chromosome has been detected. Cell division processes or G2/M phase are active at about mid of cell cycle or 12 hours incubation period, and 2 copies or 4n chromosome has been observed. The study found that most of the cell population stopped at G0/G1 phase after EPMC treatment for 12 hours while the untreated control sample developed to G2/M phase. Besides, 24 and 48 hours incubation duration allowed CL-6 cells to complete 1 and 2 cell division cycles by most cells presented in G0/G1 phase whereas most of EPMC exposed sample found at the G2/M phase. Cell cycle arrest occurs in response to DNA damage (Wenzel and Singh, 2018). Mechanism of cell cycle arrest at G0/G1 is p53-dependent, resulting in inhibition of the synthesis of DNA, cyclin-dependent kinase 4 (CDK4) and cyclin D (García-Reyes et al., 2018, Liu et al., 2003). P53-dependent arrested the transition of cells from G2 to M phase and -independent pathways through inhibition of cyclin B and Cdc2 proteins which are involved in mitosis consequences such as inhibition of mitotic spindle, centromere, or cytokinesis (Stark and Taylor, 2006; Wenzel and Singh, 2018). 

EPMC exposure at concentrations of IC_25_ and IC_50 _for 24 and 48 hours induced apoptosis in CL-6 cell line according to the nuclear envelope permeability and cell membrane alteration with the appearance of phosphatidylserine (PS) and expression of caspase 3/7. Apoptosis is a final effect of caspases 3 and 7 which are cysteine proteases activated by the initiator caspase 8 via death receptor in the extrinsic pathway or caspase 9 by DNA damage in the intrinsic pathway (McIlwain et al., 2013). Caspases 3 and 7 exert almost the same action during apoptosis, but caspase 3 is clearly related to plasma membrane blebbing, whereas caspase 7 is also involved in inflammation process (Tomita, 2017; Zhang et al., 2018). Both cell cycle arrest and apoptosis are downstream consequences of p53 protein (Chen, 2016). EPMC exposure may induce DNA damage leading to cell cycle arrest and activate caspase 3/7 expression results in cell apoptosis. 

Several anticancer agents derived from medicinal plants fail for treatment because it is the substrate of P-glycoprotein and the situation becomes worse if the compound can induce *mdr-1* gene expression (Barthomeuf et al., 2005; Lopes-Rodrigues et al., 2016). Results of the study showed that even CL-6 and Caco-2 have different EPMC sensitivity, but it does not effect on *mdr-1 *expression in both cell lines. This suggests low potential of cholangiocarcinoma cells to develop resistance to EPMC. Furthermore, the compound may has *mdr-1* expression inhibition activity in CL-6 cell as decreasing of *mdr-1* gene expression was observed in all compound exposed samples (Figure 5). Co-administration of p-glycoprotein inhibitor together with medicinal plant anticancer is an interesting option to improve cancer treatment efficacy. Also, investigation of EPMC on *mdr-1* expression mechanisms will expand knowledge for treatment innovation (Santos and Paulo, 2013). 

In summary, EPMC exerted low to moderate cytotoxic activity against cholangiocarcinoma through cell cycle arrest and cell apoptosis without inducing of *mdr-1* expression. Therefore, the compound has suitable biological activities for further development to be used as an adjunct therapy.
